# Consumer vulnerability and well-being across contexts: Implications for international businesses

**DOI:** 10.1016/j.heliyon.2023.e14612

**Published:** 2023-03-16

**Authors:** Paulo Duarte, Marcelo Augusto Linardi, Helena Sá Domingues, Susana C. Silva

**Affiliations:** a NECE - Research Centre in Business Sciences - Universidade da Beira Interior, Avenida Marquês D’Ávila e Bolama, 6201-001, Covilhã, Portugal; b Católica Porto Business School - Universidade Católica Portuguesa & CEGE, Rua Diogo Botelho 1327, 4169-005, Porto, Portugal

**Keywords:** Consumer vulnerability, Well-being, Consumer behaviour, Pandemic, Covid-19, Comparative study, Portugal, Brazil, International business

## Abstract

This article assesses the relationship between consumer vulnerability (CV) and well-being (WB) by comparing the effects of ordinary (non-pandemic) and pandemic consumption contexts among Portuguese and Brazilian consumers. Data on pre-and post-pandemic perceived vulnerability and well-being from a cross-cultural sample of 397 consumers were analyzed through structural equations modelling using the PLS-Path. The results revealed an inverse relationship between CV and well-being, which worsened with the emergence of the pandemic. Refund Policies, Product Promotions and Purchase Ability are the dimensions of CV identified as the most affected by the COVID-19 outbreak. Furthermore, fear proved to mediate the effect of vulnerability on well-being partially. The findings allow us to conclude that the most disrupted CV dimensions during COVID-19 are Refund Policy (RP), Purchase Ability (PA), and Product Promotion (PP). Studies comparing consumer vulnerability in international contexts are scarce. By finding the most critical dimensions of CV during a pandemic crisis, this study provides novel insights for companies and public institutions when planning responses and strategies to future disruptive occurrences. The conclusions represent an original contribution by analysing and comparing consumers' vulnerability in an everyday consumption situation and an extreme situation deployed by the COVID-19 pandemic. Valuable insights for governments and policymakers are provided. Firms working in international markets can use the insights to adapt their business strategy as effects on well-being vary across cultures.

## 1 Introduction

The pandemic of COVID-19 has brought unexpected challenges to populations and governments in the quest to preserve the *status quo* and have the least impact on people’s well-being. To support these challenges, many studies in the social sciences sought to understand human behaviour in the face of this outbreak, such as in understanding the adoption of preventive measures [1,2], cooperation and trust in society [3] and positive thinking [4].

The rapid international diffusion of COVID-19 has also promoted changes in buying behaviour worldwide, with unexpected repercussions on business management, marketing and public policy [[5], [6], [7]]. Several attention-grabbing phenomena have been observed among the unusual behaviours, such as the surprising worldwide toilet paper shortage. Some examples are the sudden spike in demand for essential protection items like face masks and alcohol-based hand sanitisers; and the rising number of fraud victims who bought fake preventive and healing products [[7], [8], [9]]. These widespread phenomena urge analysis and explanation to measure the pandemic’s overall impact on the CV to provide insights for companies and public institutions. According to Crockett and Grier [10], policy research should help illustrate the economic implications of this phenomenon that exploits and worsens long-standing existent disparities between people, whether this happens within a country [11] or across countries. Despite the inherently global nature of the pandemic, its size and impact have been shown to differ across countries according to the demographic characteristics, and consumer-related public policies followed.

However, consumers and companies had to adapt to the new situation and arrange coping strategies with greater or lesser restrictions. Therefore, it would be essential to understand the differentiated effects on countries with comparable cultural backgrounds yet different in size, attitudes, and behaviour towards the pandemic.

Brazil shares cultural traits with Portugal; however, its geographical dimension, location, climate, and economic and social development level vary significantly. Furthermore, despite the cultural proximity [12], there are clear differences in COVID-19 effects and lockdown impositions and prevention measures since the pandemic, making these two realities worth investigating. As of November 12, 2021, Brazil had 21.9 million cases of COVID-19 (10307.4 per 100 k persons), while Portugal had 1.1 million cases (10707.5 per 100 k persons). As a result, the total cumulative deaths per 100 k persons in Brazil (287) is 1.6 times higher than in Portugal (177.1) [13]. Considering the unbalanced situation between the two countries, which share many cultural traits, Brazil’s pandemic’s effects on consumer well-being are expected to emerge more severe. However, the poor living conditions in Brazil, the increased vulnerability, and the Brazilians' stress-free way of looking at life compared to the Portuguese can positively reduce the pandemic's negative effect on consumer well-being. In this context, this article proposes a CV model that can help understand the context's impact on consumer behaviour before and after the pandemic by comparing Portuguese and Brazilian consumers.

Applying the Conceptual Model for Consumer Vulnerability (CMCV) by Baker et al. [14] to a particular consumption context confirms and expands the original model’s capacity and scope to new research fields. The current research also aims to understand the role of CV’s dimensions proposed by Shi et al. [15] (e.g., product knowledge, product promotion, social pressure, refund policy, marketing and emotional pressure, distinguish ability, and purchase ability) in influencing consumers' behaviours in a situation where consumers' vulnerability may differ. Unquestionably, COVID-19 has increased consumer vulnerability, but it is unclear how it affected consumer decision-making and well-being. Therefore, the current study fills the gap in the literature concerning the COVID-19 impact on consumer vulnerability and well-being. In addition, assessing consumer vulnerability after the emergence of the COVID-19 pandemic will enable governments and businesses to design better and more informed strategies to decrease vulnerability and increase people's well-being.

The main objective of this article is to assess how COVID-19 impacted the relationship between consumer vulnerability (CV) and well-being (WB) by comparing the ordinary (non-pandemic) and pandemic consumption behaviours among Portuguese and Brazilian consumers. Accordingly, two research questions are proposed: How does COVID-19 impact consumer vulnerability? Can cultural contexts increase consumers' vulnerability by limiting the ability to make rational purchasing decisions detrimental to well-being?

## 2 Consumer vulnerability

The reasons behind consumers' vulnerability have been studied through a narrowed perspective, directly pointing out population-specific characteristics as the leading cause for its manifestation. Several readings focus on showing the direct relationship between specific demographic indicators, such as age [[16], [17], [18]], income [19,20], race [10], literacy levels [21,22] and consumer vulnerability. Some consumers are presented as being more prone to become victims of vulnerability during purchasing activities as their characteristics make them less capable of buying the combination of goods and services that ultimately maximises their satisfaction and, consequently, their utility [23]. However, a growing body of literature [14,[24], [25], [26]] suggests that it is inappropriate to point out individual characteristics as the unique reason for vulnerability. According to Baker et al. [14], CV results from the interaction between internal characteristics and the external environment in a consumption experience. Therefore, regardless of their characteristics, all consumers may, at some point, experience vulnerability when making economic decisions [14,15,27].

The leading conceptual model defining CV [14] proposes that vulnerability results from consumer states, external conditions, and the consumption context besides individual characteristics. The factors stemming from CV experiences should thus be considered through a holistic approach considering both internal and external dynamic influences. For this reason, vulnerability should be perceived as situational since it results from several stimuli appearing at a particular place and moment. Consequently, unlike preliminary studies suggested, a consumer who would not otherwise be classified as vulnerable can temporarily become so due to specific internal conditions and external circumstances, making CV a non-permanent state [28]. Therefore, it seems adequate to study the influence of the COVID-19 outbreak as the external stimulus or influence on the consumer’s decisions and assess its impact on well-being. Since CV is also presented as context-dependent, contrasting different contexts appears essential for fully understanding the stimulus’s true power.

### 2.1 Consumer vulnerability and well-being

Empirical evidence suggests an inverse relationship between CV and consumer WB. As a result, the greater the vulnerability experienced by a consumer, the smaller the ability to make rational purchasing decisions favouring their interests [15]. Therefore, vulnerable consumers allegedly experience an increased chance of decreasing their well-being by making unsuitable, mistaken, or misinformed purchases [16,19,21,29]. Based on all the above, it is proposed that:

H1 In ordinary or non-pandemic consumption contexts, there is an inverse relationship between consumers' vulnerability and well-being.

### 2.2 Consumer vulnerability, context, and well-being

Shi et al*.* [15] refer that the CV should be analyzed considering the environment where it is found. Due to the massive impact of the coronavirus pandemic [30], consequences are expected to elicit changes in consumers' vulnerability. Table 1, presents the significant expected impacts of COVID-19 associated with the seven dimensions of CV.

**Table 1:** Contextual analysis of the dimensions of consumer vulnerability.

Dimension	Context from Shi et al*.,* (2017)	COVID-19’s Impact
**Product Knowledge (PK)**	A consumer’s limited PK increases his chance of making mistaken, misinformed, and regretful purchases.	Limiting customers' presence inside commercial spaces only for the time strictly necessary to purchase products constraints a buyer’s ability to consider all available product alternatives and opt for the optimum one.
**Product Promotion (PP)**	PP is the capability of persuading consumers into buying particular products or services without hesitation.	Restrained mobility and lockdowns force families to stay at home, household’s TV and broadband usage has inevitably increased [31]. Consequently, consumers are subject to a greater number of advertisements than usual. This atypical exposure to product promotion influences buyers into vulnerable states, triggering irrational purchases.
**Social Pressures (SP)**	SP influences consumers towards unreasonable purchases, only to please others or avoid disagreements with others.	During February and March 2020, news around the world reported empty supermarket shelves after a spike in demand for the purchase of toilet paper (Kirk & Rifkin, 2020). Unsure of how to best act, these individuals are, therefore, likely to increase imitative behaviours.
**Refund** **Policy (RP)**	Vulnerable consumers will interpret RP as an indication that they can make carefree purchases.	Although features such as full-refund and free-cancellation policies on vacation packages [32] appear to be favouring consumers interests, literature predicts this apparent facility leads shoppers to make useless purchases that they later regret.
**Marketing and Emotional Pressures (MEP)**	The service provider’s pleasant characteristics have the power to influence consumers into making buying decisions harmful to their welfare. Furthermore, the role of emotions determinant of consumers' ability to rationally perform in the market.	Fear, worriedness, and stress are predictable emotional pressures nurtured from the coronavirus pandemic [33]. [34] goes further and explains experienced fear, anxiety, and paranoia have been influencing consumers into engaging in irrational and fraudulent purchases.
**Distinguish Ability (DA)**	DA focuses on a buyer’s capacity to detect fraudulent information and deceitful marketing methods.	
**Purchase Ability (PA)**	PA dimension assesses consumer’s power to acquire their desired options within the marketplace.	Unemployment and bankruptcy rates increased, and the sudden loss of work and shortage of income predict a general decrease in consumers buying power (Mehta et al., 2020; [35])

Considering the concept of CV, there is room for further investigation on the role of unique contexts in influencing consumers into vulnerable states. As CV impacts consumer WB [15], the current study proposes that the COVID-19 pandemic triggers higher vulnerability experiences in consumers and decreases their overall well-being. Based on this, the following hypothesis is proposed:

H2 Pandemic contexts strengthen the impact of consumer vulnerability on well-being.

By recognising the role of individual states and emotions as a basis for consumers' ability to take market decisions rationally, it is believed that the greater the emotional pressures on consumers, the greater their vulnerability [15]. Furthermore, recent findings support the ability of health risks as predictors of CV (31). Therefore, fear of health risks (i.e., Fear of COVID-19) is likely to mediate the effect of CV on well-being. Based on this, it is proposed in H3 that the greater the Fear of COVID-19, the more significant the negative impact on consumers' well-being.

H3 In pandemic contexts, fear of health risk mediates the relationship between consumers' vulnerability and well-being.

Supported by Shi et al*.* [15] findings, consumers are expected to experience different grades of vulnerability according to the specific CV dimensions due to the context. Therefore, the following hypothesis is proposed:

H4 Pandemic context impacts the seven dimensions of consumer vulnerability differently.

Two studies were developed to answer the research questions and test the proposed hypotheses. The first study targets consumer vulnerability in the ordinary (non-pandemic) context, and the second addresses the pandemic context, comparing the outcome of the increased vulnerability on well-being among Portuguese and Brazilian consumers.

## 3 Study 1: consumer vulnerability in a normal context

Study 1 is the baseline study, and its purpose is to assess and validate earlier findings by Ref. [15], proposing that CV negatively impacts consumer well-being and test hypothesis H1.

### 3.1 Design and procedures

An online survey was devised and made available in December 2020 to Portuguese and Brazilian residents to evaluate consumers' vulnerability perception in a normal or standard (i.e., non-pandemic) consumption context (Appendix A). To measure the effect of vulnerability on consumer well-being, respondents were stimulated to think about their pre-pandemic experiences and rate items adapted from the BBC Well-being Scale [36] and the CV scale proposed by Shi et al*.* [15] (see appendix A). The items were scored using a 7-point Likert scale where one stands for strongly disagree and seven represents strongly agree. Due to the study's exploratory nature and sample size, Smart-PLS 3.3 was selected to evaluate the research model [37].

Data from the two independent samples were collected, and 397 responses were obtained using a non-probabilistic sampling technique. From these, 209 consumers lived in Portugal and 188 in Brazil. The majority (68.3%) of the individuals belonged to the active working-class population aged 21 to 49. In addition, the sample is particularly highly educated, as 81% of respondents had at least concluded a bachelor’s degree. Interestingly, 54 respondents reported that their average monthly income decreased due to the pandemic.

Table 2 summarises the sample’s descriptive statistics.

**Table 2:** Sample characteristics.

		TOTAL			PORTUGAL			BRAZIL	
		*n*	%		*n*	%		*n*	%
**Gender**									
female		154	38.8		53	25.4		101	53.7
male		242	61.0		155	74.2		87	46.3
not informed		1	0.3		1	0.5		0	0
**Age**									
under 17 years		1	0.3		0	0		1	0.5
18–20 years		20	5.0		6	2.9		14	7.4
21–29 years		107	27.0		41	19.6		66	35.1
30–39 years		74	18.6		38	18.2		36	19.1
40–49 years		90	22.7		55	26.3		35	18.6
50–59 years		73	18.4		52	24.9		21	11.2
above 60 years		32	8.1		17	8.1		15	8.0
**Education**									
primary school		2	0.5		0	0		2	1.1
high school		73	18.4		41	19.6		32	17.0
bachelor’s degree		88	22.2		10	4.8		78	41.5
undergraduate degree		145	36.5		106	50.7		39	20.7
master’s degree		64	16.1		42	20.1		22	11.7
PhD or higher		25	6.3		10	4.8		15	8.0
**Occupation during COVID-19**									
Student		46	11.6		22	10.5		24	12.8
Self-employed		116	29.2		43	20.6		73	38.8
Employee		196	49.4		131	62.7		65	34.6
Unemployed		25	6.3		8	3.8		17	9.0
Retired		12	3.0		5	2.4		7	3.7
Disabled		2	0.5		0	0.0		2	1.1
**Monthly Income during COVID-19**									
under 500€		66	16.6		26	12.4		40	21.3
501€–1000€		85	21.4		47	22.5		38	20.2
1001€–1500€		78	19.6		63	30.1		15	8.0
1501€–2000€		57	14.4		35	16.7		22	11.7
over 2000€		111	28.0		38	18.2		73	38.8

**Fig. 1:**
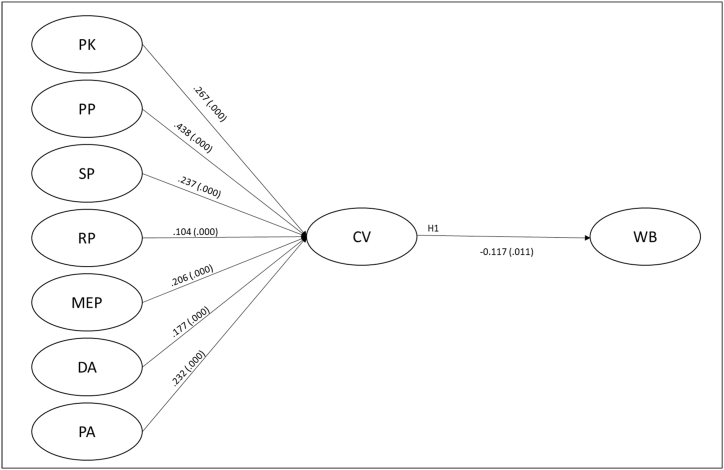
Structural model before COVID-19’s emergence.

The formative nature of the CV scale and the aim to develop incremental knowledge in CV theory demanded using the partial least squares technique (PLS-SEM) to analyse the data. Furthermore, the asymmetry of observable indicators in the dimensions of the CV scale required the use of the two-step approach for formative second-order constructs [38]. Finally, computing latent variables scores for each of the first-order variables (PK, PP, SP, RP, MEP, DA, and PA) allowed the seven dimensions of Shi et al*.* [15] to be considered predictors of the dependent variable consumer vulnerability (see Fig. 1).

A first assessment of the outer model reflective first-order variable discriminant validity, construct reliability, and convergent validity was conducted for the model’s adequate evaluation. Appropriate adjustments were made based on the initial results, and the variable MEP2, which impaired the reliability and validity of the measurement model, was excluded, as detailed in the results section. After, the adjusted model latent variable scores were calculated and used to evaluate the formative second-order construct’s reliability and validity and the final structural path model analysis.

### 3.2 Results

In PLS-Path analysis, the evaluation of the reliability and validity of the measures in the outer model is processed differently depending on each construct’s formative or reflective nature [39]. As the indicators for WB and the seven dimensions of consumer vulnerability are reflective, individual item reliability, internal consistency, and discriminant validity were assessed. After the first round of tests, the MEP2 indicator was excluded. It had a small, non-significant loading and affected the construct’s reliability, convergent validity (AVE), and discriminant validity. Although other indicators presented loadings below the benchmark value, they were not excluded as their corresponding construct had high-reliability scores (AVE>0.5 and CR > 0.7) (see Table 3) [40]. Accepting these less-than-optimal indicators aimed to keep the original scale as unchanged as possible to avoid potential bias and preserve comparability. Similarly, despite the well-being’s AVE score being below the recommended 0.5 [41], no WB indicators were excluded to ensure the model’s significance. Besides, the result was close to the minimum acceptable value (AVE_well-being_ = 0.403).

**Table 3:** Construct reliability and validity before COVID-19's emergence.

Construct	Item	Loadings	AVE	CR
Distinguish Ability	DA1	0.930	0.861	0.925
	DA2	0.925		
Marketing & Emotional Pressures	MEP1	0.747	0.553	0.787
	MEP3	0.738		
	MEP4	0.744		
Purchase Ability	PA1	0.593	0.538	0.821
	PA2	0.783		
	PA3	0.741		
	PA4	0.799		
Product Knowledge	PK1	0.548	0.519	0.842
	PK2	0.759		
	PK3	0.797		
	PK4	0.729		
	PK5	0.741		
Product Promotion	PP1	0.769	0.547	0.874
	PP2	0.845		
	PP3	0.856		
	PP4	0.827		
	PP5	0.443		
	PP6	0.604		
Refund Policy	RP1	0.637	0.694	0.869
	RP2	0.920		
	RP3	0.910		
Social Pressures	SP1	0.793	0.547	0.827
	SP2	0.787		
	SP3	0.718		
	SP4	0.650		
Well-being	WB1	0.602	0.403	0.862
	WB2	0.530		
	WB3	0.322		
	WB4	0.669		
	WB5	0.641		
	WB6	0.758		
	WB7	0.766		
	WB8	0.744		
	WB9	0.292		
	WB10	0.788		

The Fornell and Larcker [41] and the Heterotrait-Monotrait Ratio (HTMT) [42] were used to evaluate discriminant validity. The results for the Fornell and Larcker criterion shown in Table 4 confirm the discriminant validity.

**Table 4:** Discriminant validity of the adjusted model before COVID-19's emergence.

Constructs	1	2	3	4	5	6	7	8
1. Distinguish Ability	**0.928**							
2. Marketing and Emotional Pressures	0.276	**0.743**						
3. Purchase Ability	0.232	0.237	**0.733**					
4. Product Knowledge	0.428	0.146	0.301	**0.720**				
5. Product Promotion	0.175	0.394	0.242	0.202	**0.740**			
6. Refund Policies	0.071	0.243	0.096	−0.062	0.197	**0.833**		
7. Social Pressures	0.082	0.316	0.170	0.149	0.405	0.014	**0.739**	
8. Well-being	−0.065	0.000	−0.158	−0.159	0.021	0.114	−0.198	**0.635**

*Notes:* Diagonal values are the square root of the AVE between the construct and its measures. Shaded grey results are scores that did not reveal significant correlations, the other correlations are significant at 1%.

Furthermore, the second-order formative construct’s nomological validity was assessed using the adjusted model path coefficients significance [39]. All the relationships between CV and the remaining constructs revealed statistically significant *p*-values. We conclude that this latent variable portrays its desired meaning. The next step involved evaluating the inner model’s integrity and predictive ability. The results support hypothesis 1 by confirming that, in ordinary purchasing contexts, CV reveals a significant adverse effect on consumers' WB (β = −0.117, *p-value* = 0.011) (see Fig. 1).

The model predicts that for each 1° of vulnerability experienced, consumers lose −0.117 well-being during ordinary consumption contexts. However, it is observed that the variance explained (R^2^ = 0.014) and the strength of this relationship (*f*^2¨^ = 0.014) present small values, indicating that, before the emergence of the COVID-19 pandemic, the negative impact of CV on well-being was real but weak.

### 3.3 Discussion

The first study provides empirical evidence to confirm the direct, even weak, inverse relationship between CV and WB. Additionally, it settles the first quantitative evidence on the estimated size of such an effect through a structural equation model focused on predicting the WB based on CV. These findings support the formerly conceptualised correlation between the constructs suggested by Shi et al*.* [15] and reinforce the indication that the greater the vulnerability experienced by a consumer, the smaller their ability to make rational purchasing which does not harm his WB [14,21]. The empirical results show that PP (λ = 0.438, *p* = 0.000) and PK (λ = 0.267, *p* = 0.000) present the most significant contributions to CV experience.

## 4 Study 2: consumer vulnerability in pandemic

Study 2 examines the relationship between CV and consumer WB in the COVID-19 pandemic context. In addition, it seeks to assess whether the perceived Fear of COVID-19 plays a significant role in mediating this relationship.

### 4.1 Design and procedures

In this study, two new measures focused on assessing the impact of the pandemic on CV were presented to the respondents from study 1 (Appendix A). At the end of each sub-section, consumers were asked to consider the current pandemic scenario by rating a new statement formulated by authors, using a 7-point Likert scale, one being strongly disagree and seven being strongly agree. Seven new statements were introduced to assess the global change in perception due to the COVID-19 consumption scenario (Appendix B). This procedure avoided boring the respondents by asking them to answer the same 28 original items again. The pandemic weighting factors statements in Appendix B were intended to verify how consumers perceived the outbreak scenario had affected each of the seven dimensions of CV. This approach enabled predicting the effects of the pandemic context on each of the different CV dimensions. These seven new variables were used as “weighting factors”, reflecting respondents' perception of the influence of COVID-19 on each of the seven dimensions of vulnerability. Using the weighting factors, a proxy for the latent variables scores of CV after the emergence of COVID-19 was estimated. For that purpose, it was assumed that a person who scored one (i.e., totally disagree) to a particular dimension’s weighting factor statement perceived that the pandemic did not affect that dimension. As such, this consumer’s scores for that latent variable’s items will be the same as those awarded during the evaluation of the ordinary consumption context, thus conveying an equal level of vulnerability. The procedure was applied to all dimensions, and new scores for the 28 items of CV given COVID-19’s emergence were calculated using the formula:

1 PKc_i_ = PK_i_ + WF_PK_ −

Where:

PKc = Product Knowledge Dimension after the emergence of COVID-19
PK = Product Knowledge Dimension before the emergence of COVID-19
WF_PK_ = Weighting factors for the perceived impact of COVID-19
c = the item in the context of the pandemic
i = the adjusted item number

The resulting variables for each of the seven CV dimensions after the pandemic’s emergence (PKc, PPc, SPc, RPc, MEPc, DAc and PAc) ranged from 1 to 13.

Finally, to verify the mediating role of fear of health risk during the pandemic, respondents fully rated the seven Fear for COVID-19 scale items by Ahorsu et al. [43].

The Partial Least Squares (PLS) method was used to assess the new data and test the impact of CV on WB after the pandemic (H2) and evaluate the potential mediating role of Fear of Health Risk on consumers' WB (H3) in the new context. To evaluate the differences between CV levels before and after the emergence of COVID-19 (H4), a comparison was conducted between the weights of the CV dimensions and path coefficients between CV and WB in study 1 and study 2.

### 4.2 Results

**Fig. 2:**
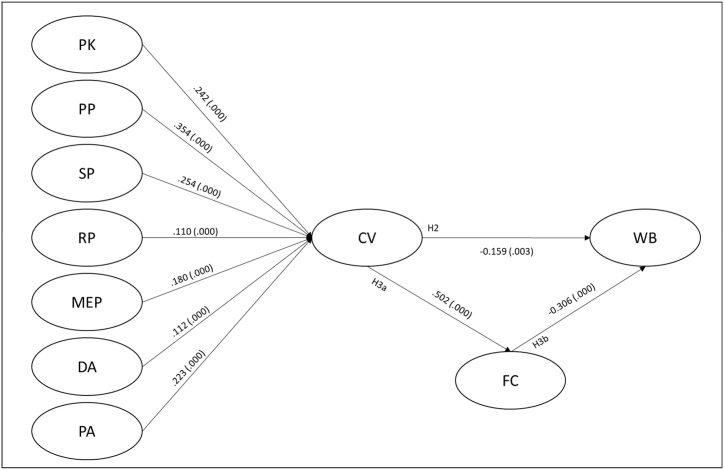
Structural model after COVID-19’s emergence.

An adjusted model was built based on the two-step approach for formative second-order constructs. The analysis was started by replicating in the post-COVID-19 context the first steps in study 1. The latent variable scores for each first-order variable (PKc, PPc, SPc, RPc, MEPc, DAc, and Pac) were calculated, as seen in Fig. 2.

The analysis of the post-COVID-19 situation required the introduction of a new latent variable addressing the Fear of COVID-19 (FC). The addition enabled further analysis of the mediating effect of fear of health risks (H3) in the relationship between CV and WB.

The initial round of tests revealed that the post-COVID-19 model did not require any adjustments, unlike the former. However, the MEP2 indicator was also excluded to guarantee comparability between both studies. All indicators revealed scores above the suggested thresholds regarding the model’s internal consistency and convergent validity evaluation (Table 5). Table 6 validates the measurement model’s discriminant validity.

**Table 5:** Construct reliability and validity after COVID-19’s emergence.

Construct	Item	Loadings	AVE	CR
Distinguish Ability	DA1c	0.975	0.951	0.975
	DA2c	0.975		
Marketing & Emotional Pressures	MEP1c	0.922	0.852	0.945
	MEP3c	0.919		
	MEP4c	0.928		
Purchase Ability	PA1c	0.882	0.844	0.956
	PA2c	0.933		
	PA3c	0.927		
	PA4c	0.931		
Product Knowledge	PK1c	0.854	0.819	0.958
	PK2c	0.916		
	PK3c	0.934		
	PK4c	0.912		
	PK5c	0.906		
Product Promotion	PP1c	0.936	0.865	0.975
	PP2c	0.956		
	PP3c	0.953		
	PP4c	0.946		
	PP5c	0.873		
	PP6c	0.912		
Refund Policy	RP1c	0.905	0.891	0.961
	RP2c	0.963		
	RP3c	0.962		
Social Pressures	SP1c	0.938	0.887	0.969
	SP2c	0.954		
	SP3c	0.948		
	SP4c	0.928		
Well-being	WB1c	0.704	0.549	0.923
	WB2c	0.611		
	WB3c	0.833		
	WB4c	0.832		
	WB5c	0.806		
	WB6c	0.792		
	WB7c	0.756		
	WB8c	0.642		
	WB9c	0.778		
	WB10c	0.609		
Fear of COVID-19	FC1	0.735	0.591	0.910
	FC2	0.777		
	FC3	0.732		
	FC4	0.757		
	FC5	0.771		
	FC6	0.783		
	FC7	0.824

**Table 6:** Discriminant validity of the adjusted model after COVID-19’s emergence.

	1	2	3	4	5	6	7	8	9
1. Distinguish Ability	**0.975**								
2. Fear of COVID-19	0.359	**0.769**							
3. Marketing & Emotional Pressures	0.387	0.385	**0.923**						
4. Purchase Ability	0.349	0.291	0.427	**0.919**					
5. Product Knowledge	0.419	0.273	0.253	0.407	**0.905**				
6. Product Promotion	0.290	0.377	0.421	0.338	0.314	**0.930**			
7. Refund Policies	0.115	0.193	0.287	0.276	0.117	0.232	**0.944**		
8. Social Pressures	0.309	0.421	0.547	0.359	0.305	0.543	0.190	**0.942**	
9. Well-being	−0.260	−0.439	−0.212	−0.237	−0.269	−0.162	−0.281	−0.258	**0.741**

*Notes:* Diagonal values are the square root of the AVE between the construct and its measures. All variables revealed significant correlations.

Like study 1, in the pandemic context, the second-order formative constructs supported nomological validity [39]. All relationships between CV after COVID-19 and the remaining constructs revealed significant *p*-values. (See Appendix C).

All hypotheses were supported, and the path analysis results suggest that the outbreak intensifies the significant negative relationship between CV and WB (See Table 7).

**Table 7:** Structural relationship after the emergence of COVID-19.

Hypothesis	Coefficient	*f*^*2*^	SD	*t*-value	*p*-value	R^2^	Decision
**H2:** CVc -> WBc	−0.159	0.024	0.054	2.937	0.003	0.212	Supported
**H3:** CVc -> FC -> WBc	−0.180	0.024	0.031	5.864	0.000	0.212	Supported
**H3a:** CVc -> FC	0.502	0.337	0.038	13.283	0.000	0.252	Supported
**H3b:** FC -> WB	−0.360	0.123	0.049	7.308	0.000	0.212	Supported

According to the model results, during the pandemic context, for each 1° of vulnerability experienced, consumers lose 0.159 of well-being. The R^2^ value further suggests that, in such a scenario, 21.2% of WB variation is explained by CV. Although larger than the effect predicted during an ordinary consumption context, the *f*^2^ still portrays a small size effect from CV on WB according to Cohen’s [44] criteria. This result suggests that the emergence of the COVID-19 pandemic has intensified the negative impact of vulnerability on consumers' WB. Furthermore, the results indicate a partial mediation through Fear of COVID-19 (H3) (See Table 7). The model predicts that, during pandemic contexts, for each 1° of vulnerability experienced, consumers lose 0.180 of WB if they also experience Fear of COVID-19, meaning that the negative effect of CV on WB is more significant for consumers who display higher fear for the pandemic.

The analysis of the differences in the weights of the CV dimensions shows that all dimensions changed in the post-COVID-19 context (See Table 8). Social Pressure and Refund Policy have increased their importance in CV formation, where Product Promotion showed the topmost decrease.

**Table 8:** Consumer vulnerability dimensions - context analysis.

CV Dimensions	Pre-COVID-19		Post- COVID-19		Mann-Whitney		
	Mean	SD	Mean	SD	U	Z	*p*-value
PK	0.267	0.011	0.242	0.009	8457.5	−25.525	0.000
PP	0.438	0.014	0.354	0.009	0.0	−27.376	0.000
SP	0.237	0.011	0.255	0.009	28805.5	−21.070	0.000
RP	0.104	0.005	0.110	0.005	43876.0	−17.788	0.000
MEP	0.206	0.008	0.180	0.006	612.5	−27.246	0.000
DA	0.177	0.008	0.112	0.004	0.0	−27.384	0.000
PA	0.232	0.010	0.224	0.008	68244.0	−12.435	0.000

### 4.3 Discussion

Study 2 extends the findings of study 1 by confirming the negative impact of COVID-19 on the relationship between vulnerability and consumer well-being. This significant heightened impact may be associated with the impacts of the pandemic on consumers' emotional states, which had already been shown to be a driver of CV [45]. The introduction of the FC endorses these findings. The correlation inspection shows that FC has a positive relationship with all dimensions of CV. Moreover, the partial mediation by FC of the relationship between CV and WB confirms the impact of emotions in the loss of rationality and the increased vulnerability. This finding demands extra attention from public policymakers to impose programmes devoted to protecting the population segments more likely to feel vulnerable [46].

The differences in the factor weights of CV formation pre- and post-COVID-19 were evaluated to improve understanding of the phenomenon.

## 5 Robustness testing

To confirm the robustness of the results, a set of control tests and multi-group models were assessed. The robustness tests sought to verify whether the propositions from the conceptual model could suffer significant changes according to the cultural context and characteristics of the samples.

Control variables are designed to rule out alternative answers to those considered in the study. In general, they influence the dependent and independent variables but are not the object of the study. The differentiating aspect of the control variables is that they are external to the model and not related to the hypotheses being tested [47]. In the current study, following Bernerth and Aguinis [48] considerations, it was assessed whether there would be a need to introduce control variables. This inclusion was considered necessary since no study on vulnerability has considered somewhat disadvantaged consumers, such as the young [16], the elderly [49,50], and the disabled [46]. Therefore, considering the data available, it was considered worthy of testing the influence of age as the control variable.

Baker et al*.* [14] characterise CV as a transitory state arising from the interaction of their characteristics with the marketing environment. Based on that assertion, it is possible to assume that more remarkable cognitive ability contributes to lower consumer vulnerability. There are also studies relating vulnerability to education (e.g. Refs. [21,46]). Thus, education was also included as a control variable, and its influence on vulnerability was tested.

Given the complexity of the model and not contaminating the model, eventually generating multicollinearity with the inclusion of foreign variables, in the current study, we have opted for conducting a correlation analysis of the control variables with the scores of the higher-order latent variables. The results in Table 9 show that, in general, there is no significant correlation between the control variables and Consumer Vulnerability in pre- or post-COVID-19 contexts, except for education.

**Table 9:** Control Variables – Kendall's tau-b.

Variable	Age	Education	Gender	Professional status	Income
CV	-0.031	-.083∗	0.041	0.033	-0.003
CVc	-0.024	-.114∗∗	0.002	0.026	-0.027

∗ p < .05.

∗∗ p < .01.

An invariance analysis was also performed to assure robustness by assessing if the proposed model results and its psychometric properties are similar for both populations, thus strengthening the reliability of the conclusions. The results in Table 10 do not reveal significant differences in the construct factor loadings for the higher-order variable Consumer Vulnerability, except for the SP, which shows a low difference (*delta* = −0.047, *p-value* = 0.005).

**Table 10:** Invariance model testing – structural model.

Paths	Coefficient			p-Value
	Brazilians	Portugueses	Diference	
CVc -> Fc	0.482	0.526	0.043	0.556
CVc -> WBc	−0.148	−0.195	−0.048	0.654
Fc -> WBc	−0.227	−0.481	−0.254	**0.011**
DAc -> CVc	0.111	0.118	0.008	0.388
MEPc -> CVc	0.183	0.181	−0.003	0.824
PAc -> CVc	0.230	0.222	−0.008	0.626
PKc -> CVc	0.243	0.250	0.007	0.694
PPc -> CVc	0.357	0.351	−0.006	0.744
RPc -> CVc	0.107	0.115	0.008	0.405
SPc -> CVc	0.270	0.223	−0.047	**0.005**

Appendix D reports a detailed analysis showing that of the 71 measurement items of the model, only three items (Fc5, PA4c, WB7c) showed significant differences. Therefore, considering that the small number of items with differences is small compared to the total number of items, we feel confident that the measurement model behaves equivalently across the two populations.

## 6 Discussion and implications

This study, which aims to assess the relationship between Consumer vulnerability and well-being before and after the COVID-19 pandemic, confirms the direct inverse relationship between CV and WB. Furthermore, it supplies the first quantitative evidence on the estimated effect of the relationship supporting the previously conceptualised correlation between both constructs suggested by Ref. [15]. Therefore, the findings reinforce the assertion that the greater the vulnerability experienced by a consumer, the smaller their ability to make rational purchasing, having their well-being in mind when making a consumption decision [14,21].

### 6.1 Theoretical implications

The findings highlight the power of unique contexts in running consumers into vulnerable states [14,45]. Studies 1 and 2 show that worldwide pandemic scenarios influence CV and WB levels, reinforcing the need for public interventions in such complex scenarios to minimise consumers' feelings of vulnerability and prevent further consequences, namely psychological disorders. Precisely, the findings predict that the negative effect of CV on WB is more significant during pandemic contexts. The emergence of a global outbreak increases the variation in WB, explained by consumers' level of vulnerability. The level of fear consumers mediates this relationship as high levels of fear enlarge the negative effect of CV on WB. These findings stress the role of individual states and emotions as a basis of consumers' ability to perform rationally in the market, supporting [14]. Moreover, it calls for considering the need to design strategies and specialised programs to improve consumers' comprehension of their emotions and be prepared to deal with them in stressful consumption contexts.

While former findings had determined health risk as a predictor of CV [51], the present study adds to this understanding by confirming that fear of health risk partially mediates the relationship between CV and WB in a pandemic context. Furthermore, it is found that the negative effect of vulnerability on WB is greater for consumers who fear the pandemic than those who do not. This finding recommends that companies increase investments in more effective communication plans to enhance their value proposals during pandemic contexts. Additionally, according to the Extended Parallel Process Model (EPPM) [[52], [53], [54]] fear is often used as a trigger to push behavioural changes. It can be considered in public policy campaigns to highlight the psychological risks involved in engaging with a particular behaviour. For instance, carelessness or rushed purchase decisions, without proper comparison between different channel members or brand options, since it complies with the belief that individuals, through fear, are more inclined to adopt a specific behaviour.

Nevertheless, it is recognised that in some circumstances and for some people, fear is not a trigger [53]. Therefore, the threat may not necessarily lead to the desired response [54]. In this case, policymakers should carefully judge how to deal with fear, as emphasising it too much may cause the consumer to freeze, which can be even more harmful.

The findings allow us to conclude that the most disrupted CV dimensions during COVID-19 are Refund Policy (RP), Purchase Ability (PA), and Product Promotion (PP). By finding the most critical dimensions of CV during a pandemic crisis, this study has valuable insight for public institutions when planning their responses and strategies in possible future outbreaks. Based on the current findings, policymakers may want to consider acting over the offer side by enforcing companies to take standards that ensure enhanced levels of protection for consumers during distressing times. These conclusions are particularly valuable as the literature has shown that governmental and administrative strategies to prevent virus spread should not proliferate CV at any level [55].

The analysis of the significance of socio-demographic factors in influencing CV indicates that the manifestation of vulnerability is influenced by internal and external dynamic factors [14]. It can be concluded that Portuguese consumers are more elastic to changes in the dimensions of CV than Brazilians under a typical purchasing environment. Besides, Portuguese consumers also appear to be more sensitive to changes in the dimensions directly influencing their level of CV and WB than those living in Brazil. Interestingly, under pandemic contexts, the negative effect of Fear of COVID-19 on WB was significantly lower for Brazilian consumers. This may be a consequence of the “Emergency Aid” program by the Brazilian Senate that approved an emergency aid of US$ 116 (R$ 600) to informal workers and US$ 232 (R$ 1200) to mothers responsible for supporting the family. However, Brazilian consumers perceive a more significant impact of the pandemic on their vulnerability. It is puzzling to conclude that Brazilians understand to be more vulnerable, but the fear was not so impactful. A possible cause of this effect is the normalised fragility with which the population faces daily survival challenges. High rates of violence and a failing public health system may be anaesthetising causes of this diminished perception of the fear of covid and its impact on well-being. A recent survey found that only 11% of Brazilians interviewed were extremely worried about their health during the COVID-19 epidemic [2]. This paradox deserves further attention in future studies. Identifying the most significant detractors of CV in routine situations and scenarios of greater emotional destabilisation supported by the framework proposed by Shi et al*.* [15] opens new opportunities for further academic research. The study findings represent an original contribution to the literature as it analyses and compare consumers' vulnerability in a normal day-by-day consumption situation and in the confused situation deployed by the COVID-19 pandemic, which was absent in the existing body of knowledge.

### 6.2 Managerial or public policy implications

The findings show that negative emotional states significantly influence the CV and demand consumers to redouble attention in these situations. In this context, they may consider creating digital solutions that ensure consumers understand the whole process and do not make hasty decisions to avoid promoting non-rational and rushed decisions. Avoidance of advantage-taking situations is crucial to ensure consumers' confidence, promote long-term relationships, and adopt a better use of technology to favour the consumer. Nowadays, banks and insurance firms can accurately predict each client’s credit risk or claim. Efforts to recognise and adjust the sales process to each customer’s degree of vulnerability would undoubtedly bring more outstanding suitability and stability.

Consumers are recommended to have redoubled effort and use additional resources in situations of high variability in their emotional states, where non-rational purchases prevail. Therefore, consumer protection institutions should be empowered. Even though the economy is slowing down, the demand for protection is growing. The consumer seems more aware of the means at their disposal to control commercial practices, especially for essential products. Consumers should also take an active position in this process by increasing their vigilance against predatory practices and adopting a more conscious consumption behaviour in line with their needs. They should avoid overstocking and denouncing bad practices and endorsing companies conducting good practices.

By assessing country-specific realities, the results enable companies to develop strategies that best suit consumers in each country in future potential outbreaks and regular consumption situations. Companies should focus on channel design strategies to lighten consumers' vulnerabilities. Managers may go through the customer journeys to identify how the imposed security restrictions may have restricted elders' ability to access product information to find ways of providing guidance to ensure that customers make rational purchasing decisions that do not harm their well-being. Companies may also instruct their digital influencers to promote conscious and informed shopping by providing clear communication and avoid fake promoting fake news. Finally, Companies should ensure that they comply with safety standards and only advertise certified and safe products.

For lawmakers, this research is relevant to stress the importance of protecting the consumer as it empirically confirms the concerns of Baker et al*.* [14] regarding CV’s harms. During the pandemic, many instances of legal enforcement of lockdown practices were employed to ensure law enforcement and the consequent limitation of some individual rights. However, as far as laws focused on protecting consumer rights under odd consumption contexts are concerned, their reality was quite different, with only a few cases of specific regulations being noticed [56]. For example, specific labour regulations during the pandemic can be set to help companies face the adverse context and help them to preserve jobs. Legislators have a long journey to create norms and specific laws for pandemic contexts to reduce the power imbalance between companies and consumers to protect the most vulnerable consumers and secure their emotional states. Price protection, limiting the number of units bought by sale and consumer, and intensifying preventive communication are just a few examples of actions firms can take to reduce Fear and CV. Capital charge relief is another important measure that governments can take. Lending and credit support through capital charge relief to loans for real estate and retail purchases would positively impact consumer vulnerability and well-being. However, steadiness and correct use of the threat is required as well. Particularly in Brazil, this did not happen. Instead, companies were given opportunities to (re)act. Citizens were exposed to frequent changes in health authorities. The government often changed its position with higher contamination numbers, adding instability to an already fearful, complex, and unpredictable context. On the other hand, extreme fear mobilisation is a double-sword effect on consumers in this respect. So, cautiousness and planning seem essential to be applied by policymakers and governments.

## 7 Limitations and future research direction

One limitation that must be accounted for is the sample size and composition. The number of respondents from Brazil and Portugal is not balanced, which may have influenced the results. Thus, an individual country analysis should be prioritised when assessing the conclusions. Furthermore, it should be noted that as 81% of the sample hold a college degree, which may have impacted the results. The current study used the conceptual Model for Consumer Vulnerability (CMCV) by Baker et al. [14] and the scales by Shi et al. [15]. Future studies may use a different theoretical framework which could address the validity issues of the CV scale by Shi et al*.* [15] by proposing a more objective scale. Besides, as this research proves that the current CV scale cannot be applied to different contexts, it would also be interesting that the new scale would not require additional weighting factors for CV assessment during different contexts.

Regarding future directions, upcoming studies could try to answer other questions regarding the consequences of an increased vulnerability for consumers and international businesses, namely, how to protect consumers, especially those displaying higher fear levels, or how to anticipate market abuses arising from extraordinary situations such as pandemics. Besides, specific governmental policies and business strategies that triggered CV experiences during a pandemic could also be examined to assess their impact on national and international business, providing further support to current findings.

## Author contribution statement

Paulo Duarte, PhD; Susana Silva, PhD: Conceived and designed the experiments; Analyzed and interpreted the data; Contributed reagents, materials, analysis tools or data; Wrote the paper.

Marcelo Augusto Linardi, MSc; Helena Sá Domingues, PhD: Conceived and designed the experiments; Performed the experiments; Analyzed and interpreted the data; Contributed reagents, materials, analysis tools or data; Wrote the paper.

## Funding statement

Support from NECE – Research Centre in Business Sciences funded by the Multiannual Funding Programme of R&D Centres of FCT – Fundação para a Ciência e a Tecnologia, under the project UIDB/04630/2020 and CEGE – Research Centre in Management and Economics, funded by the Multiannual Funding Programme of R&D Centres of FCT – Fundação para a Ciência e a Tecnologia, under the project UIDB/00731/2020 are gratefully acknowledged.

## Data availability statement

Data will be made available on request.

## Declaration of interest’s statement

The authors declare that they have no known competing financial interests or personal relationships that could have appeared to influence the work reported in this paper.

## Figures and Tables

**Appendix A:** Questionnaire constructs and Indicators

Construct and Items
Consumer Vulnerability - Shi et al*.* 2017
Product Knowledge
PK1: When buying a product, I do not usually consider the potential harm it will PK2: generate during the process of use.
PK2: When buying a product, I am not sure whether it will be harmful to me.
PK3: When buying a product, I do not always compare it with other similar products.
PK4: When buying a product, I do not always know similar products from other brands.
PK5: I do not have a specific evaluation criterion when buying products.
Product Promotion
PP1: I often buy advertised products.
PP2: I often buy products recommended in the current advertisement.
PP3: I often make buying decisions according to what product information has been promoted through the mass media.
PP4: I often make buying decisions among similar products according to what product attributes have been demonstrated through the mass media.
PP5: I often buy products that are recommended in the market promotion activity.
PP6: I will buy a product without hesitation if the spokesperson of it is a familiar celebrity to me.
Social Pressure
SP1: I often buy a product in order to please others.
SP2: I often buy products in order to cater to others.
SP3: I often buy a product in order to avoid disagreement with others.
SP4: When buying a product, I often look at the other’s face.
Refund Policy
RP1: When buying a product, if there is an invalid refund guarantee, I will make the decision without hesitation.
RP2: When buying a product, if there is an unconditional refund guarantee, I will make the decision without hesitation.
RP3: When buying a product, if there is a product assurance or refund agreement, I will make the decision without hesitation.
Marketing and Emotional Pressure
MEP1: I always change my decisions under the persuasion of the salesperson.
MEP2: Hospitable service will always make me buy more products.
MEP3: I often feel embarrassed to refuse a salesman’s recommendation.
MEP4: My buying decisions are often influenced by emotions.
Distinguish Ability
DA1: When buying a product, I usually do not know what information is fraudulent.
DA2: Within the consumption process, I usually could not tell which marketing method was fraudulent.
Purchase Ability
PA1: I often buy a product that is similar to what I want at a lower price.
PA2: When buying a product, I often have no alternative but to give up my first preference and choose the poorer one.
PA3: When buying a product, I often realise that there are very few options within my ability.
PA4: I often could not get what I want and had to buy a similar substitute.
**Well-being - Kinderman et al. 2011**
WB1: Do you feel depressed or anxious?
WB2: Do you feel that you are able to enjoy life?
WB3: Do you feel in control over your life?
WB4: Do you feel able to live your life the way you want?
WB5: Do you feel able to do the things you choose to do?
WB6: Are you satisfied with your physical health?
WB7: Are you satisfied with your ability to perform your daily living activities?
WB8: Are you satisfied with your ability to work?
WB9: Are you satisfied that you have enough money to meet your needs?
WB10: Are you satisfied with your access to health services?
**Fear of COVID-19 - Ahorsu et al. 2020**
FC1: I am most afraid of coronavirus-19.
FC2: It makes me uncomfortable to think about coronavirus-19.
FC3: My hands become clammy when I think about coronavirus-19.
FC4: I am afraid of losing my life because of coronavirus-19.
FC5: When watching news and stories about coronavirus-19 on social media, I become nervous or anxious.
FC6: I cannot sleep because I am worried about getting coronavirus-19.
FC7: My heart races or palpitates when I think about getting coronavirus-19.

**Appendix B:** Pandemic Weighting Factors

Statement
WPK: The current epidemic context of COVID-19 has conditioned my ability to obtain information about products before their acquisition.
WPP: The current epidemic context of COVID-19 has reinforced the role of communication strategies in my purchasing decisions.
WSP: The current epidemic context of COVID-19 has intensified the role of social pressures in my purchasing decisions.
WRP: The current epidemic context of COVID-19 influenced the delivery, exchange, return and order cancellation, making me give up on a purchase.
WMEP: The current epidemic context of COVID-19 has deepened the role of emotional pressures in my purchasing decisions.
WDA: The current epidemic context of COVID-19 has limited my ability to distinguish fraudulent business strategies.
WPA: The current epidemic context of COVID-19 has reduced and conditioned my ability to purchase the desired products.

**Appendix C:** Nomological Validity Test for Second-Order Formative Constructs

Context	Relationship	Coefficient	SD	*t*-Value	*p*-value
**A** (before COVID-19’s emergence)	DA -> CV	0.505	0.034	14.721	0.000
	MEP -> CV	0.622	0.029	21.299	0.000
	PA -> CV	0.558	0.035	15.803	0.000
	PK -> CV	0.560	0.041	13.499	0.000
	PP -> CV	0.777	0.022	34.997	0.000
	RP -> CV	0.262	0.052	5.028	0.000
	SP -> CV	0.575	0.035	16.211	0.000
	WB -> CV	−0.117	0.049	2.383	0.018
**B** (after COVID-19’s emergence)	DAc -> CVc	0.555	0.038	14.679	0.000
	MEPc -> CVc	0.699	0.027	26.307	0.000
	Pac -> CVc	0.678	0.027	25.237	0.000
	PKc -> CVc	0.626	0.033	18.892	0.000
	PPc -> CVc	0.777	0.020	37.943	0.000
	RPc -> CVc	0.394	0.041	9.602	0.000
	SPc -> CVc	0.754	0.021	35.642	0.000
	WBc -> CVc	−0.339	0.048	7.079	0.000
	FC -> CVc	0.502	0.035	14.342	0.000

**Appendix D:** Invariance Model Testing – Measurement Model

Items		Outer Loadings			*p*-value
		Brazilians	Portugueses	Diference	
DA1c <- DAc		0.980	0.972	−0.008	0.210
DA1c <- CVc		0.527	0.569	0.042	0.627
DA2c <- DAc		0.976	0.975	−0.001	0.861
DA2c <- CVc		0.482	0.603	0.121	0.175
FC1 <- Fc		0.698	0.762	0.064	0.201
FC2 <- Fc		0.776	0.783	0.007	0.878
FC3 <- Fc		0.731	0.736	0.005	0.924
FC4 <- Fc		0.717	0.787	0.069	0.202
FC5 <- Fc		0.828	0.723	−0.105	**0.010**
FC6 <- Fc		0.741	0.813	0.071	0.166
FC7 <- Fc		0.820	0.830	0.010	0.791
MEP1c <- MEPc		0.926	0.917	−0.009	0.542
MEP1c <- CVc		0.711	0.597	−0.114	0.091
MEP3c <- MEPc		0.922	0.913	−0.009	0.586
MEP3c <- CVc		0.683	0.602	−0.080	0.193
MEP4c <- MEPc		0.944	0.913	−0.031	0.051
MEP4c <- CVc		0.677	0.565	−0.112	0.093
PA1c <- PAc		0.904	0.862	−0.042	0.070
PA1c <- CVc		0.570	0.586	0.016	0.833
PA2c <- PAc		0.946	0.918	−0.028	0.071
PA2c <- CVc		0.614	0.611	−0.003	0.956
PA3c <- PAc		0.937	0.916	−0.021	0.185
PA3c <- CVc		0.648	0.621	−0.027	0.666
PA4c <- PAc		0.948	0.910	−0.039	**0.007**
PA4c <- CVc		0.625	0.655	0.030	0.672
PK1c <- PKc		0.867	0.841	−0.027	0.347
PK1c <- CVc		0.506	0.650	0.144	0.075
PK2c <- PKc		0.917	0.915	−0.002	0.912
PK2c <- CVc		0.596	0.633	0.037	0.630
PK3c <- PKc		0.943	0.927	−0.016	0.175
PK3c <- CVc		0.551	0.633	0.082	0.325
PK4c <- PKc		0.907	0.918	0.011	0.562
PK4c <- CVc		0.514	0.592	0.078	0.356
PK5c <- PKc		0.917	0.897	−0.020	0.287
PK5c <- CVc		0.504	0.645	0.141	0.079
PP1c <- PPc		0.926	0.943	0.017	0.233
PP1c <- CVc		0.691	0.741	0.050	0.413
PP2c <- PPc		0.956	0.952	−0.004	0.687
PP2c <- CVc		0.739	0.725	−0.014	0.822
PP3c <- PPc		0.950	0.953	0.003	0.762
PP3c <- CVc		0.731	0.718	−0.013	0.837
PP4c <- PPc		0.946	0.943	−0.002	0.866
PP4c <- CVc		0.725	0.711	−0.015	0.809
PP5c <- PPc		0.878	0.862	−0.016	0.516
PP5c <- CVc		0.633	0.706	0.073	0.260
PP6c <- PPc		0.915	0.905	−0.009	0.583
PP6c <- CVc		0.701	0.747	0.047	0.419
RP1c <- RPc		0.913	0.893	−0.019	0.443
RP1c <- CVc		0.238	0.378	0.140	0.206
RP2c <- RPc		0.966	0.960	−0.005	0.602
RP2c <- CVc		0.313	0.408	0.095	0.398
RP3c <- RPc		0.971	0.953	−0.018	0.108
RP3c <- CVc		0.313	0.429	0.116	0.285
SP1c <- SPc		0.936	0.928	−0.009	0.598
SP1c <- CVc		0.702	0.661	−0.041	0.444
SP2c <- SPc		0.951	0.950	−0.001	0.931
SP2c <- CVc		0.735	0.676	−0.059	0.260
SP3c <- SPc		0.950	0.936	−0.013	0.483
SP3c <- CVc		0.754	0.676	−0.078	0.139
SP4c <- SPc		0.931	0.913	−0.018	0.431
SP4c <- CVc		0.732	0.669	−0.064	0.274
WB10c <- WBc		0.524	0.685	0.161	0.063
WB1c <- WBc		0.675	0.717	0.042	0.576
WB2c <- WBc		0.619	0.609	−0.010	0.885
WB3c <- WBc		0.836	0.830	−0.006	0.842
WB4c <- WBc		0.791	0.853	0.062	0.138
WB5c <- WBc		0.804	0.813	0.009	0.898
WB6c <- WBc		0.797	0.794	−0.003	0.903
WB7c <- WBc		0.835	0.698	−0.137	**0.030**
WB8c <- WBc		0.681	0.607	−0.074	0.311
WB9c <- WBc		0.837	0.741	−0.096	0.056
